# Prevalence of renal impairment and use of nephrotoxic agents among patients with bone metastases from solid tumors in the United States

**DOI:** 10.1002/cam4.403

**Published:** 2015-02-08

**Authors:** Jorge Arellano, Rohini K Hernandez, Sally W Wade, Kristina Chen, Melissa Pirolli, David Quach, Jane Quigley, Alexander Liede, Vahakn B Shahinian

**Affiliations:** 1 Amgen Inc.Thousand Oaks and South San Francisco, California; 2 Wade Outcomes Research and ConsultingSalt Lake City, Utah; 3 IMS HealthPlymouth Meeting, Pennsylvania; 4 Department of Internal Medicine, Kidney Epidemiology and Cost Center, University of MichiganAnn Arbor, Michigan

**Keywords:** Bone metastasis, chronic kidney disease, intravenous bisphosphonates, renal impairment, solid tumor

## Abstract

The renal status of patients with bone metastases secondary to solid tumors and their treatment with nephrotoxic agents is not well characterized. This retrospective study analyzed electronic medical records data from US-based oncology clinics to identify adult (age ≥18) solid tumor patients with first bone metastasis diagnosis and ≥1 serum creatinine recorded between January 1, 2009 and December 31, 2013. Patients with multiple myeloma, multiple primary tumor types, acute renal failure, and/or end-stage renal disease were excluded. Using the Chronic Kidney Disease Epidemiology Collaboration formula, we determined the prevalence of renal impairment (RI: single estimated glomerular filtration rate [eGFR] value <60 mL/min per 1.73 m^2^) and chronic kidney disease (CKD: ≥2 eGFR values <60, at least 90 days apart). We also examined the use of intravenous bisphosphonates (IV BP) and other nephrotoxic agents. Approximately half of the 11,809 patients were female. Breast (34%) and lung (28%) tumors were the most common. At bone metastasis diagnosis, mean age was 67 years and 24% of patients exhibited RI. The 5-year prevalence was 43% for RI and 71% for CKD among RI patients. Nearly half (46%) of CKD patients received IV BP in the 12 months following their confirming eGFR and 13% of these patients received at least one other nephrotoxic agent during that period. This is the first US-based study to examine the prevalence of RI among patients with bone metastases from solid tumors. RI is common at bone metastases diagnosis, and a substantial proportion of patients develop RI or CKD as their disease progresses. Whenever possible, treatments that are potentially less damaging for the kidney should be considered for patients with or predisposed to RI.

## Introduction

There is growing interest in the intersection between kidney disease and cancer, with some professional organizations, such as the American Society of Nephrology as well as the Cancer and the Kidney International Network (C-KIN), even exploring the development of an “onconephrology” subspecialty 1,2. The relationship between kidney disease and cancer is complex. Chronic kidney disease (CKD) is associated with an increased risk of some cancers, and cancer itself can contribute to the development of CKD or acute kidney injury 1,3. Furthermore, the presence of CKD is associated with an increased risk of death among patients with cancer, and many agents used for the treatment of cancer (or complications from cancer, e.g., antibiotics, antifungals) are potentially nephrotoxic 1,3–15.

Patients with cancer complicated by bone metastases may be particularly at risk for renal impairment (RI) or CKD 16,17. Pamidronic and zoledronic acids are intravenously administered bisphosphonates (IV BP) that are commonly used to prevent bone complications in these patients. While these agents offer therapeutic benefit, they are also associated with deterioration of renal function, which limits or, at a minimum complicates, treatment choices in individuals concurrently treated with other nephrotoxic agents (e.g., chemotherapy) or otherwise predisposed to RI 16,17.

The renal status of patients with bone metastases secondary to solid tumors, and use of nephrotoxic agents among these patients has not been well characterized. We, therefore, estimated the prevalence of RI in US patients with bone metastases secondary to solid tumors. Electronic medical records (EMR) data from oncology clinics were critical for the conduct of this study since EMR capture results from laboratory studies (i.e., serum creatinine values) that are routinely ordered in that setting. In addition, these EMR allowed us to examine use of nephrotoxic agents, including IV BP, in patients with evidence of RI.

## Methodology

This study was conducted with EMR data housed in the Oncology Services Comprehensive Electronic Records (OSCER) database. OSCER includes outpatient data for a representative sample of more than 569,000 cancer patients treated at 565 community and hospital-affiliated oncology clinics from 2004 forward. Patients reside in all 50 states and all payer types are represented (commercial, Medicare, Medicaid, self-pay, and other). Patient records in OSCER are deidentified and fully compliant with the Health Insurance Portability and Accountability Act (HIPAA) of 1996.

During each oncology clinic visit, detailed data including ICD-9-CM (International Classification of Diseases Classification 9th Revision Clinical Modification) diagnosis codes, CPT-4 (current procedural terminology) procedure codes, laboratory test results, and treatments administered or prescribed are captured, along with the relevant service dates, in the EMR. Laboratory test dates, results, applicable units and normal reference ranges are typically entered directly into the EMR.

For this study, we identified adult (age ≥18) solid tumor patients with a first diagnosis of bone metastasis and at least one serum creatinine recorded between 1 January 2009 and 31 December 2013. Patients with ICD-9-CM diagnosis codes for multiple myeloma, multiple primary tumor types, acute renal failure, and/or end stage renal disease on or before their first bone metastasis diagnosis were excluded.

The Chronic Kidney Disease Epidemiology Collaboration (CKD-EPI) formula was used to compute the estimated glomerular filtration rate (eGFR) from serum creatinine values recorded in the EMR 18. Race is a required input for this formula and was defaulted to Caucasian when missing or unknown (19% of the population). The strict definition of CKD requires a reduced GFR for at least 3 months. Therefore, the term “renal impairment” is used if a patient had only a single occurrence of eGFR <60 mL/min per 1.73 m^2^. CKD is used for patients with at least two eGFR values <60 mL/min per 1.73 m^2^ measured at least 90 days apart. Lists of nephrotoxic agents (including IV BP, chemotherapy, biologic therapy, and targeted therapy) and anticancer agents were predefined based on literature review and expert clinical consultation. Results are reported overall, and in some cases by tumor type: breast, prostate, lung, renal or other.

The demographic and clinical characteristics of the study population, including mean eGFR and renal status based on assessments in the 3 months prior to and 1 month after bone metastasis diagnosis, were summarized. We estimated the prevalence of RI and of CKD in 2009–2013 for the study population overall and stratified by tumor type. We also examined distribution of these patients across eGFR categories: <15, 15–29, and 30–59 mL/min per 1.73 m^2^. Among patients with RI, we determined the number and proportion using any nephrotoxic agents, IV BP, and IV BP plus another nephrotoxic agent in the 12 months prior to and 12 months after the lowest recorded eGFR. Among CKD patients, use of these agents was assessed in the 12 months prior to and 12 months after the date of the confirming (second) eGFR value.

## Results

**Figure 1:**
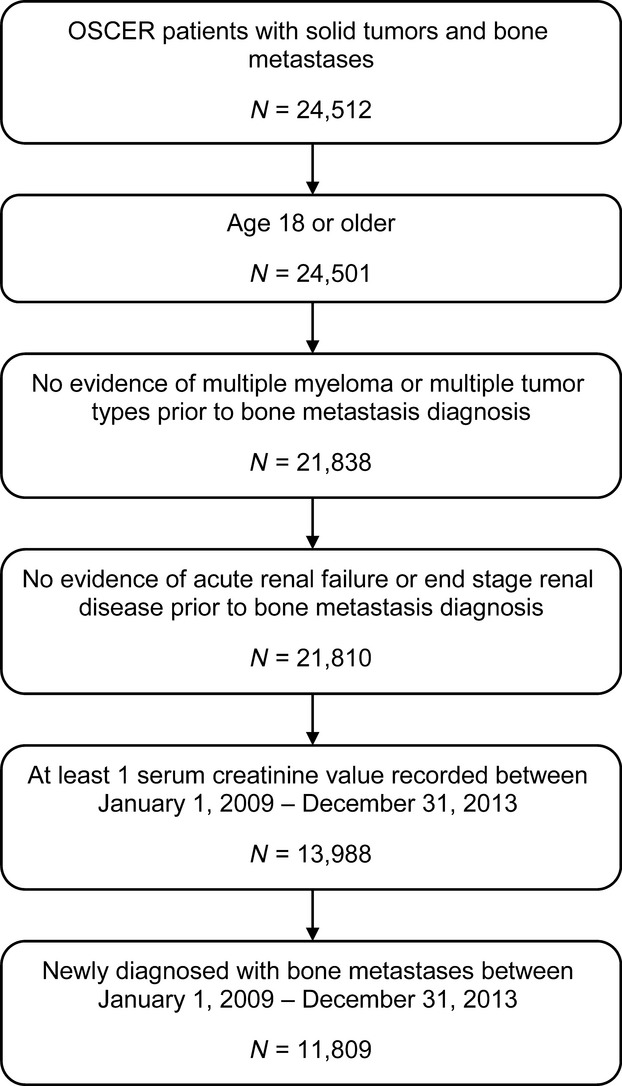
Patient selection.

A total of 24,512 patients with diagnoses of solid tumors and bone metastasis were identified. Among these patients, 11,809 (48%) met the inclusion criteria. The criterion for patients to have at least one serum creatinine value recorded during the study period eliminated ∼36% of the patients who met the criteria applied earlier in the selection process (Fig.1).

Demographic and clinical characteristics of the study population are presented in Table1. Females comprised just over half of the study population (51%), and the majority (66%) of the patients were Caucasian. On average, patients were 67 years old at the time of their bone metastasis diagnosis.

**Table 1:** Characteristics of study patients (*N* = 11,809)

Characteristics	*N*	%
Mean age in years at time of bone met diagnosis	66.9	NA
Mean age in years during reporting period^1^	67.0	NA
Female	6023	51.0
Race/ethnicity		
African American	1095	9.3
Asian	78	0.7
Caucasian	7798	66.0
Hispanic	38	0.3
Other	515	4.4
Unknown/not available	2285	19.4
Tumor type		
Breast	3968	33.6
Prostate	2772	23.5
Lung	3241	27.5
Other solid tumors	1828	15.5
Use of any anticancer drug within 12 months before bone metastasis diagnosis	3268	27.7
Use of any nephrotoxic drug within 12 months before bone metastasis diagnosis	2760	23.4
eGFR within 3 months prior to and 1 month after bone metastasis diagnosis		
Number of patients with eGFR	11,189	94.8
Mean eGFR	77.3	
eGFR range		
eGFR ≥90	3590	32.1
eGFR 60 to <90	4925	44.0
eGFR 30 to <60	2397	21.4
eGFR 15 to <30	240	2.1
eGFR <15	37	0.3
Use of any nephrotoxic drug therapy during study period^1^	8140	68.9
Use of any IV BP during study period^1^	6402	54.2
Use of zoledronic acid during study period^1^	6217	52.7
Use of pamidronic acid during study period^1^	271	2.3

eGFR, estimated glomerular filtration rate (mL/min per 1.73 m^2^); IV BP, intravenous bisphosphonate.

1 On or after bone metastasis diagnosis; study period: 1 January 2009–31 December 2013.

Breast and lung cancer were the most common tumors, accounting for 34% and 28% of the study population, respectively. Of the 11,189 (95%) patients with a serum creatinine assessment around the time of bone metastasis diagnosis, the mean eGFR was 77.3 mL/min per 1.73 m^2^ and 24% exhibited RI (eGFRs <60 mL/min per 1.73 m^2^). Approximately one-quarter (2760) of patients used a nephrotoxic agent in the 12 months prior to the bone metastasis diagnosis and 69% of patients used such agents at any time thereafter. Among those using nephrotoxic agents after their bone metastasis diagnosis, IV BP use was observed in 6402 patients (79%). Zoledronic acid was the most commonly used nephrotoxic agent (77%), followed by carboplatin (31%), bevacizumab (16%), and gemcitabine (16%) (Table S1).

**Figure 2:**
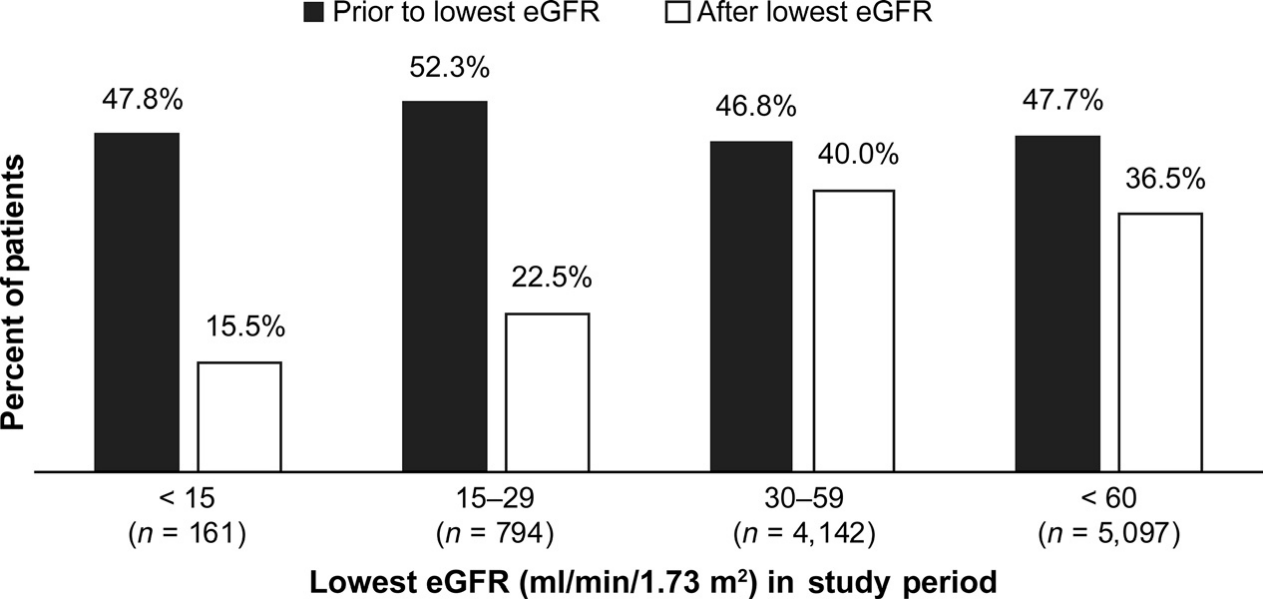
Intravenous bisphosphonates use in patients with renal impairment (*N* = 5097).

RI was common among patients with bone metastases, with 43% of patients having at least one eGFR <60 mL/min per 1.73 m^2^ during the 5-year study period (Table2). In the majority (81%) of patients with RI, the lowest eGFR was between 30 and 59 mL/min per 1.73 m^2^. IV BP use appeared to decrease after RI was observed. Nearly half (48%) of patients with RI used IV BP in the 12 months prior to their lowest eGFR compared with 37% in the 12 months following their lowest eGFR. This utilization pattern was observed in all three eGFR categories (<15, 15–29, 30–59), with the most marked decrease in IV BP use (an absolute drop of 30–32 percentage points) among patients in the lowest two categories (Fig.2).

**Table 2:** Prevalence of renal impairment and use of nephrotoxic agents

	Lowest eGFR (mL/min per 1.73 m^2^) in study period							
	<15		15–29		30–59		<60	
	*N*	%	*N*	%	*N*	%	*N*	%
Patients by eGFR category^1^	161	1.4	794	6.7	4142	35.1	5097	43.2
Received any nephrotoxic agent(s) prior to lowest eGFR^2^	100	62.1	526	66.3	2642	63.8	3268	64.1
Received any nephrotoxic agent during 12 months after lowest eGFR^2^	41	25.5	288	36.3	2203	53.2	2532	49.7
Received both IV BP and another nephrotoxic agent during 12 months after lowest eGFR^2^	9	5.6	46	5.8	491	11.9	546	10.7

eGFR, estimated glomerular filtration rate; IV BP, intravenous bisphosphonate.

1 Percentage of all study patients (*N* = 11,809).

2 Percentage of all patients with at least one eGFR <60 mL/min per 1.73 m^2^ (*N* = 5097).

A similar trend was observed in the use of any nephrotoxic therapy, including IV BPs, with the percentage of patients using these agents decreasing from 64% to 50% after RI was detected (Table2). As with IV BP use alone, larger decreases in use of nephrotoxic agents were observed among patients with the greatest levels of RI.

Approximately 70% (8247) of patients in the study population had at least two eGFRs 90 days or more apart (Table3). CKD prevalence was 35% overall (76% among patients with renal tumors; 31% to 38% among patients with other tumor types, Table4) and 71% among the 4258 patients with RI and two eGFRs at least 90 days apart. Before the confirming eGFR, 56% of CKD patients received IV BP, compared with 46% in the 12 months afterwards (Table3). Similarly, 72% of CKD patients received at least one nephrotoxic agent (including IV BP) prior to confirmation, compared with 59% of patients afterwards. Among the 1687 patients who received any nephrotoxic agent after CKD was confirmed, 13% received both an IV BP and another nephrotoxic agent.

**Table 3:** Prevalence of CKD and use of nephrotoxic agents

	Confirming^1^ eGFR (mL/min per 1.73 m^2^) in study period							
	<15		15–29		30–59		<60	
	*N*	%	*N*	%	*N*	%	*N*	%
Patients by eGFR category^2^	26	0.3	203	2.5	2636	32.0	2865	34.7
Received any IV BP prior to confirming eGFR^3^	9	34.6	80	39.4	1519	57.6	1608	56.1
Received any IV BP during 12 months after confirming eGFR^3^	2	7.7	39	19.2	1289	48.9	1330	46.4
Received any nephrotoxic agent prior to confirming eGFR^3^	11	42.3	111	54.7	1944	73.8	2066	72.1
Received any nephrotoxic agent during 12 months after confirming eGFR^3^	4	15.4	67	33.0	1616	61.3	1687	58.9
Received both IV BP and another nephrotoxic agent during 12 months after confirming eGFR^3^	0	0.0	9	4.4	362	13.7	371	13.0

CKD, chronic kidney disease; eGFR, estimated glomerular filtration rate; IV BP, intravenous bisphosphonate.

1 Second eGFR <60 mL/min per 1.73 m^2^.

2 Percentage of all patients with at least two eGFR values at least 90 days apart (*N* = 8247).

3 Percentage of all patients with CKD (*N* = 2865).

**Table 4:** Prevalence of CKD and use of nephrotoxic agents by tumor type

	Tumor type									
	Breast (*N* = 3225)		Prostate (*N* = 2133)		Lung (*N* = 1826)		Renal (*N* = 190)		Other (*N* = 873)	
	*N*	%	*N*	%	*N*	%	*N*	%	*N*	%
Patients with CKD^1^	1076	33.4	811	38.0	559	30.6	144	75.8	275	31.5
Received any IV BP prior to confirming eGFR^2^	707	65.7	419	51.7	307	54.9	57	39.6	118	42.9
Received any IV BP during 12 months after confirming eGFR^2^	565	52.5	361	44.5	249	44.5	48	33.3	107	38.9
Received any nephrotoxic agent prior to confirming eGFR^2^	793	73.7	431	53.1	511	91.4	97	67.4	234	85.1
Received any nephrotoxic agent during 12 months after confirming eGFR^2^	622	57.8	366	45.1	422	75.5	83	57.6	194	70.6
Received both IV BP and another nephrotoxic agent during 12 months after confirming eGFR^2^	111	10.3	8	1.0	164	29.3	19	13.2	69	25.1

CKD, chronic kidney disease; eGFR, estimated glomerular filtration rate; IV BP, intravenous bisphosphonate.

1 Percentage of patients with at least two eGFR values at least 90 days apart.

2 Percentage of patients with CKD.

## Discussion

This study is the first to examine the prevalence of RI and use of nephrotoxic agents in patients with bone metastases secondary to solid tumors. Based on the presence of a single eGFR <60 mL/min per 1.73 m^2^, a recently recommended approach 19, ∼24% (2865/11,809) of patients in this large cohort exhibited RI around the time of their bone metastasis diagnosis. With this same method, the 5-year prevalence (2009–2013) of RI was 43% among these patients with bone metastases secondary to solid tumors. The prevalence of CKD was 35% among patients with at least two eGFRs available. IV BP was the most commonly used nephrotoxic agent before and after RI and CKD were detected.

In 2007, Launay-Vacher et al. published results from the Renal Insufficiency and Anticancer Medications (IRMA) study which was a large observational cohort study conducted to assess the prevalence of renal insufficiency in cancer patients in France 20. Although the patient population (all cancer patients) and elements of the study design (e.g., use of Cockcroft-Gault and abbreviated Modification of Diet in Renal Disease Improving Global Outcomes for estimating renal insufficiency [aMDRD]) differ from ours, results from the IRMA study provide some context for our findings. In IRMA's sample of 4685 patients from 15 oncology centers, results based on Cockcroft-Gault and aMDRD indicated that 57% and 53% of patients had creatinine clearance (CrCL) <90 mL per minute, and 20% and 12% had CrCL <60 mL per minute. The majority of IRMA patients were also receiving nephrotoxic anticancer agents at the time of the study; the most common anticancer drugs prescribed were 5-FU, cyclophosphamide, docetaxel, epirubicin, and gemcitabine.

The IRMA study has long served as the seminal source of prevalence data for RI in cancer patients, despite its relatively small sample size and narrow geographic focus. In the time since the original publication, the IRMA data have been further analyzed to provide estimates of renal insufficiency in specific patient subgroups including the elderly, and those with breast, prostate, and lung cancer 21–24. The Belgian Renal Insufficiency and Anticancer Medications (BIRMA) was a similar, though smaller study (1218 solid tumor patients from seven oncology clinics), which has also served as a primary source of data on the prevalence of renal insufficiency in cancer patients since its inception in 2006 25.

The medical landscape tends to change quickly over time and these changes often lead to knowledge gaps and necessitate new research. For example, since the IRMA and BIRMA studies were completed, the Cockcroft-Gault formula for estimating the glomerular filtration rate is no longer routinely used, and a new formula (CKD-EPI) has been developed. The literature notes that CKD-EPI provides a more accurate estimate of the glomerular filtration rate than the Modification of Diet in Renal Disease equation 18. CKD-EPI is currently more commonly used in clinical trials, but offers an important alternative that could replace the MDRD equation in routine clinical practice 18,26. These developments presented the opportunity to not only provide unique data on RI prevalence among patients with bone metastases related to solid tumors, but the opportunity to do so using a better estimation formula that is on the way to becoming new the gold standard. In addition, the OSCER data were not only more recent, but also provided a 5-year window during which to assess prevalence, compared with the retrospective data collected during two 15-day windows in 2004 for the IRMA study and from a 1-month window in 2006 for the BIRMA study. As noted earlier, the OSCER database provided broad geographic reach within the US and access to EMR for patients from all 50 states who received care at one of the 565 participating oncology clinics.

Cancer patients are at increased risk for RI; age, preexisting kidney disease, and chronic comorbidities (e.g., diabetes, hypertension, cardiac insufficiency, autoimmune diseases such as rheumatoid arthritis) are known risk factors 26. A number of commonly used cancer therapies may also affect renal function, since these agents are generally cleared through the kidney. These include chemotherapy agents, molecular targeted agents, pain medications, radiopharmaceuticals, and IV BP used to prevent skeletal complications in patients with bone metastases 20,25,26. In the IRMA and BIRMA study populations, between 82% and 89% of patients were receiving an anticancer drug, the majority of which were nephrotoxic agents 20,25. In our study population, only 28% of patients received any anticancer drug in the 12 months prior to their bone metastasis diagnosis, and 23% of patients received at least one nephrotoxic agent in that period. This difference may reflect differences in both the mix of tumor types in the study populations (IRMA and BIRMA: 41–42% breast, 12–13% colorectal, 8–9% lung, 6–7% ovarian/gynecologic and 5–8% prostate) and cancer stage 20,25. In any case, the percentage of patients in our study population who received nephrotoxic therapies increased dramatically from 23% to 69% following bone metastasis diagnosis, with the majority (79%) of those patients having received IV BP.

Routine assessment of renal function is an important aspect of oncology care, and such monitoring is particularly important for high-risk patients including the elderly, patients with renal tumors or predisposing comorbid conditions, and patients who are actively using nephrotoxic therapies 21,26–28. The International Society of Geriatric Oncology, for example, highlighted the complexity of managing older cancer patients and noted the importance of monitoring renal function and responding to decreases in function by adjusting or discontinuing dosing of known nephrotoxic therapies, including IV BPs 28.

Oncology care is complex and for any given patient, treatment strategies may require making difficult trade-offs between the potential benefits and side effects or complications associated with both therapeutic and supportive care agents. To illustrate this point, our study indicates that 37% of patients with RI (Fig.2) and 46% of those with CKD (Table2) received IV BP during the 12-months after RI was detected. In many cases, these patients also received another nephrotoxic agent during this same time period further increasing their risk for worsening of their renal function.

Pamidronic and zoledronic acids were approved for treatment of bone metastasis in 1996 and 2002, respectively 29. These IV BPs were the only agents available for the treatment of bone metastases until 2010 when denosumab, a fully human monoclonal antibody and RANK Ligand inhibitor, received the US Food and Drug Administration's approval for use to prevent skeletal-related events (i.e., pathologic fractures, spinal cord compressions, radiation to bone, bone surgery [SRE]) in patients with bone metastases secondary to solid tumors 30,31. Denosumab demonstrated superiority over zoledronic acid in preventing SRE in three large, randomized, controlled trials 32–34. Given that three main antiresorptive therapies have been approved for prevention of skeletal-related events, individual treatment decisions should consider the patient's unique clinical situation relative to each agent's mechanism of action and labeled side effect profile. Considerations include, for example, the potential for adverse effects on renal function among patients treated with bisphosphonates and the potential for increased risk of hypocalcaemia among patients with severe renal dysfunction treated with denosumab.

Even though our results are consistent with the limited literature on this topic, it is important to keep a few limitations in mind. While the OSCER database provides detailed clinical data for a large sample of cancer patients, these data are generally limited to services that are provided in participating oncology/hematology clinics. Therapies administered outside of these clinics would not be captured, which may result in underestimation of nephrotoxic agent treatment, including IV BPs. We also note that patients who received their bone metastasis diagnosis later in the study period had shorter follow-up, which may also result in underestimation of use of nephrotoxic agents and/or RI. Our reliance on the availability of serum creatinine values collected in the course of routine practice eliminated nearly 8000 potential patients from the study population. This was somewhat surprising as we would expect the majority of solid tumor patients, especially those with bone metastases, to receive routine renal function testing. Furthermore, this selection criterion may have biased the population toward patients who were perceived by their oncologists to be at greater risk of renal problems, and as such may have been more likely to undergo renal function testing. A recent study acknowledged that this potential bias may be exacerbated when two serum creatinine measurements are required, and the authors concluded that a single eGFR in the outpatient setting was sufficient to estimate CKD prevalence 19. To address this potential bias, we determined prevalence using both a single and a confirming eGFR, and regardless of the method used, the prevalence of RI was considerable.

In conclusion, this study provides unique data on the prevalence of RI in patients with bone metastases secondary to solid tumors. In this large patient cohort, between 2009 and 2013, the prevalence of RI was 43%, and among those with RI and multiple eGFR assessments, the prevalence of CKD was 71%. Despite the notable prevalence of RI, nephrotoxic agents, including IV BPs, were commonly used. While it is not always possible to tailor treatment choices in light of patients’ renal status, treatments that are potentially less damaging for the kidney should be considered for those with or predisposed to RI.

## Supporting Information

**Table S1.** Use of nephrotoxic agents after date of bone metastasis diagnosis.
