# Supplementary material for: Prevalence of renal impairment and use of nephrotoxic agents among patients with bone metastases from solid tumors in the United States
Source: Cancer Med. 2015 Feb 8;4(5):713–20. doi: 10.1002/cam4.403 (PMC4430264; doi:10.1002/cam4.403)
Supplement: Supplementary file 1 — Table S1. Use of nephrotoxic agents after date of bone metastasis diagnosis. [file cam40004-0713-sd1.docx]

Supplemental Table 1 Use of Nephrotoxic Agents After Date of Bone Metastasis Diagnosis

| Agent | Patients (N = 8,140) | |
| --- | --- | --- |
|  | Number | Percent |
| Zoledronic acid | 6,227 | 76.5% |
| Carboplatin | 2,546 | 31.3% |
| Bevacizumab | 1,326 | 16.3% |
| Gemcitabine | 1,278 | 15.7% |
| Pemetrexed | 1,037 | 12.7% |
| Cisplatin | 484 | 6.0% |
| Pamidronate | 271 | 3.3% |
| Oxaliplatin | 247 | 3.0% |
| Doxorubicin | 229 | 2.8% |
| Sunitinib | 122 | 1.5% |
| Sorafenib | 63 | 0.8% |
| Ifosfamide | 8 | 0.1% |
| Interferon-gamma | 8 | 0.1% |
| Interferon alfa-2b | 5 | 0.1% |
| Methotrexate | 5 | 0.1% |
| Mitomycin | 3 | <0.1% |
| Aldesleukin | 1 | <0.1% |
| Interferon alfa-2a | 1 | <0.1% |
| Interferon-alfa | 1 | <0.1% |
| Streptozocin | 1 | <0.1% |
